# Combining Next-Generation Sequencing and Immune Assays: A Novel Method for Identification of Antigen-Specific T Cells

**DOI:** 10.1371/journal.pone.0074231

**Published:** 2013-09-19

**Authors:** Mark Klinger, Katherine Kong, Martin Moorhead, Li Weng, Jianbiao Zheng, Malek Faham

**Affiliations:** Research, Sequenta Inc., South San Francisco, California, United States of America; University Paris Sud, France

## Abstract

In this study, we combined a novel sequencing method, which can identify individual clonotypes based on their unique T cell receptor (TCR) rearrangement, with existing immune assays to characterize antigen-specific T cell responses. We validated this approach using three types of assays routinely used to measure antigen-specific responses: pentamers which enable identification of T cells bearing specific TCRs, activation marker expression following antigen stimulation and antigen-induced proliferation to identify cytomegalovirus (CMV) specific clonotypes. In one individual, 8 clonotypes were identified using a pentamer reagent derived from the CMV pp65 protein. The same 8 clonotypes were also identified following sequencing of cells that upregulated an activation marker following incubation with an identical peptide derived from pp65. These 8 and an additional 8 clonotypes were identified using a more sensitive CFSE-based proliferation assay. We found clear sequence homology among some of the clonotypes identified, and the CDR3 region in one clonotype was identical to a previously published pp65-specific clonotype sequence. Many of these CMV-specific clonotypes were present at frequencies below 10^−5^ which are undetectable using standard flow-cytometric methods. These studies suggest that an immune response is comprised of a diverse set of clones, many of which are present at very low frequencies. Thus, the combination of immune assays and sequencing depicts the richness and diversity of an immune response at a level that is not possible using standard immune assays alone. The methods articulated in this work provide an enhanced understanding of T cell-mediated immune responses at the clonal level.

## Introduction

Several tools have been developed to study the T cell response in an individual, particularly in the context of disease and treatment. Many of these methodologies focus on studying T cells specific to a particular antigen. The gold standard method to detect antigen-specific T cells is the use of direct multimer staining, which requires the laborious development of specific HLA-restricted reagents. Other assays, including ELISPOT, intracellular cytokine staining, and proliferation assays, enumerate antigen-specific T cells based on detection of activation following stimulation of the T cells *in vitro* with antigen [1]–[5].

These tools, which are collectively referred to as “immune assays,” have contributed immensely to our understanding of immune responses. However, many of these methods suffer from low sensitivity which has been reported at the level of 10^−3^ or 10^−4^. Additionally, these assays provide readouts of the total immune response and are unable to specifically characterize the diversity of the component T cells, or clonotypes, that contribute to an immune response. Finally there have been reports of substantial variability in the quantitative readouts of several of these assays, particularly those relying on T cell activation [6]–[9].

Next-generation sequencing has recently emerged as a highly sensitive method for characterization of the immune repertoire [10]–[17]. In this method, individual clonotypes are identified based on their unique T cell receptor rearrangements. The approach relies on high throughput sequencing of the TCRβ repertoire, which is accomplished by sequencing regions of the variable (V), diversity (D), joining (J), and constant (C) gene segments. The sequences are analyzed to determine similar sequences that form a clonotype, whose frequency can be determined by the number of member reads. These unique clonotypes and their frequencies can then be tracked in serial samples from an individual over time. This enables the characterization of the immune repertoire diversity at the level of a single cell, which translates into assay sensitivity below 10^−6^ [16]. One drawback of this approach is that it does not necessarily identify clonotypes specific to a particular antigen. We therefore sought to combine the tremendous sensitivity of the sequencing approach with the functional information provided by the immune assays.

## Materials and Methods

### Subjects and Samples

Characterized PBMCs were purchased from Cellular Technology Limited. Cells were thawed, washed and either lysed with RLT plus buffer (Qiagen) for nucleic acid purification or cultured overnight in the presence of peptides (see below) to identify antigen-specific T cells.

### Antigen-Specific T Cell Assays, Flow Cytometry and Cell Sorting

Antigen-specific cells were identified using a variety of assays: pentamer binding, cell surface marker upregulation (CD137, CD107) following short-term peptide incubation, and proliferation following relatively long-term peptide incubation. Pentamer-specific T cells were identified by incubating PBMCs with HCMV pp65_495–504_ Pentamer (ProImmune) according to manufacturer’s instructions. The procedures for obtaining viable antigen-specific T cells based on acquisition of the cell surface markers CD137/107 (for CD8 antigen-specific T cells) following brief *in vitro* incubation with peptides have been described in detail elsewhere [18]–[23]. Briefly, complete media containing 15% Fetal Bovine Serum (FBS), non-essential amino acids, glutamine and antibiotics was used for peptide incubations. Thawed PBMCs were washed and suspended at ∼400,000 cells per well (96-well U-bottom plates) in complete media. Unconjugated antibodies directed against CD28 and CD49d were then added to the wells containing the suspended cells. Peptides derived from CMV pp65 (HCMVA (pp65) (JPT Peptide Technologies) were added directly to the cell/antibody mixture, according to manufacturer’s instructions. A single peptide derived from CMV pp65 (sequence NLVPMVATV; herein referred to as ‘pp65_495_’) was used at 2 µg/ml. pp65 ‘PepMix” and CEF+ peptide pools (JPT Peptide Technologies) were added directly to the cell/antibody mixture, according to manufacturer’s instructions. Following addition of peptides, cells were incubated at 37°C for ∼18 hours. Negative control incubations were prepared as outlined above without addition of peptides. At the end of the incubation, cells were harvested from the culture and stained with antibodies for analysis and sorting by flow cytometry. For each CD8 antigen-specific assay (CD137 and CD107), fluorescently conjugated antibodies to the following cell surface markers were used for flow cytometry: CD8, CD3 and either CD137 or CD107a and CD107b. Cells were then washed and suspended in PBS containing FBS (2%) and 4′,6-diamidino-2-phenylindole (DAPI) for exclusion of non-viable cells.

Carboxyfluorescein diacetate, succinimidyl ester (CFSE)-labeled PBMCs were incubated as outlined above for 6 days in the presence of peptide and antibodies directed against CD28 and CD49d. Antigen-specific CD8^+^ T cells were identified and sorted based on CFSE dilution at day 6.

Cells were acquired and sorted using a FACSAria (BD Biosciences) instrument. Sorted antigen-specific (CD3^+^CD8^+^CMVpentamer^+^, CD3^+^CD8^+^CD137^+^, CD3^+^CD8^+^CD107a/b^+^, or CD8^+^CFSE^low^) and non-antigen-specific (CD3^+^CD8^+^CD137^−^, CD3^+^CD8^+^CD107a/b^−^) cells were pelleted and lysed in RLT Plus buffer for nucleic acid isolation. Analysis of flow cytometry data files was performed using FlowJo (Ashland, OR).

### RNA and cDNA Preparation

RNA (and DNA) was isolated using AllPrep DNA/RNA mini and/or micro kits, according to manufacturer’s instructions (Qiagen). RNA was reverse transcribed to cDNA using Vilo kits (Life Technologies).

### TCRβ Amplification and Sequencing

cDNA was amplified using locus specific primer sets for TCRβ. This amplification reaction reproducibly amplified all possible RNA transcripts found in the sample containing the rearranged TCRβ locus regardless of which variable (V) segment and which common constant (C) region allele each rearranged molecule possessed, while appending the necessary sequences for cluster formation and sample indexing.

First stage primers were designed so as to allow for the amplification of all known alleles of the germline sequences, as described previously [16], [24]. At the 5′ ends of the V segment primers, universal sequences complementary to second stage PCR primers were appended. Primers were optimized such that each possible V and C segment was amplified at a similar rate so as to minimally skew the repertoire frequency distribution during the amplification process. Specificity of the primers was, in contrast, not optimized as the primer sequences could be mapped and removed from the eventual sequence read. Thus a given sequence may have been amplified by multiple primers.

In the second stage PCR, primers on the C side annealed to the C segment with a 5′ tail that contained the sequence primer and the P5 sequence used for cluster formation in the Illumina Genome Analyzer sequencer. Primers on the V side annealed to the V segment with a 5′ tail that contained the sequence primer and the P7 sequence used for cluster formation. For each sample, one pair of primers is used in the second stage. On the C side, it is always the same primer. On the V side, it is one of a set of primers which differs by a 6 base index. Specifically, the primers on the V side of the amplification constituted one of a set of primers, each of which had a 3′ region that annealed to the overhang sequence appended in the first reaction but which further contained one of multiple 6 base pair indices that allowed for sample multiplexing on the sequencer. Each of these primers further contained a 5′ tail with the P7 sequence used for cluster formation in the Illumina Genome Analyzer sequencer.

First stage PCR was carried out using a high fidelity polymerase (AccuPrime, Life Technologies) for 16 cycles. A second stage PCR was carried out for 22 cycles on 1/100 of the amplification products from the first stage PCR. Each sample contained a unique identifying tag. Samples were pooled and purified using the QIAquick PCR purification kit (Qiagen). Cluster formation and sequencing in both directions was carried out per the manufacturer protocol (Illumina, Inc., La Jolla, CA). Specifically, three sequencing reactions were performed. First 115 bp were sequenced from the C side sufficient to sequence through the junctional sequence from C to V. At this point, the synthesized strand was denatured and washed off. A second sequencing primer was annealed that allowed the sample index to be sequenced for 6 cycles to identify the sample. At this point the reverse complement strand was generated per the Illumina protocol. A final sequencing read of 95 bp was obtained from the V- to-C direction providing ample sequence to map the V segment accurately. The sequencing data was then analyzed to determine the clonotype sequences, as described previously [24].

### Clonotype Determination

A clonotype was defined when at least 2 identical sequence reads were obtained. Briefly, after exclusion of low quality reads, sequence data were then analyzed to determine the clonotype sequences including mapping to germline V and J consensus sequences. First, the sample index sequences were used to identify which of the sequences originate from which of the pooled samples. Sequences whose index were not a perfect match to one of the indices used in a specific run were excluded. Next the forward read was used to map the J segment. Since all the sequences started from the same position of the J segments, all the J segments started at a predefined sequencing position. The first 25 bp of the J segments were used to map the J segment. Any read with more than 5 high quality mismatches to the known J segments was excluded from further analysis.

After J segment identification, V segments were mapped. The reverse read was used for this purpose. First, the V primer was mapped and excluded. Thereafter, the next 70 bases of the reverse read were mapped to the known V segments. Reads that did not map to J and V segments were excluded. The next step in mapping involved identifying the frame that related the forward and reverse reads and this allowed a continuous sequence from J to V to be constructed. This was done using the last 15 bases of the forward read which were reliably within the V segment regardless of NDN length. While these bases could be of relatively lower sequence quality as they were at the terminal end of a long read, they could be used to map within a single identified V segment in order to identify the position at which the two reads could be joined. Finally, the known V and J sequences to which the reads map were used to identify the point in the forward read at which the sequences at the junctions diverged from these mapped segments.

### Analytical Methods: Analysis Details and Clonotype Selection Criteria

**Figure 1:**
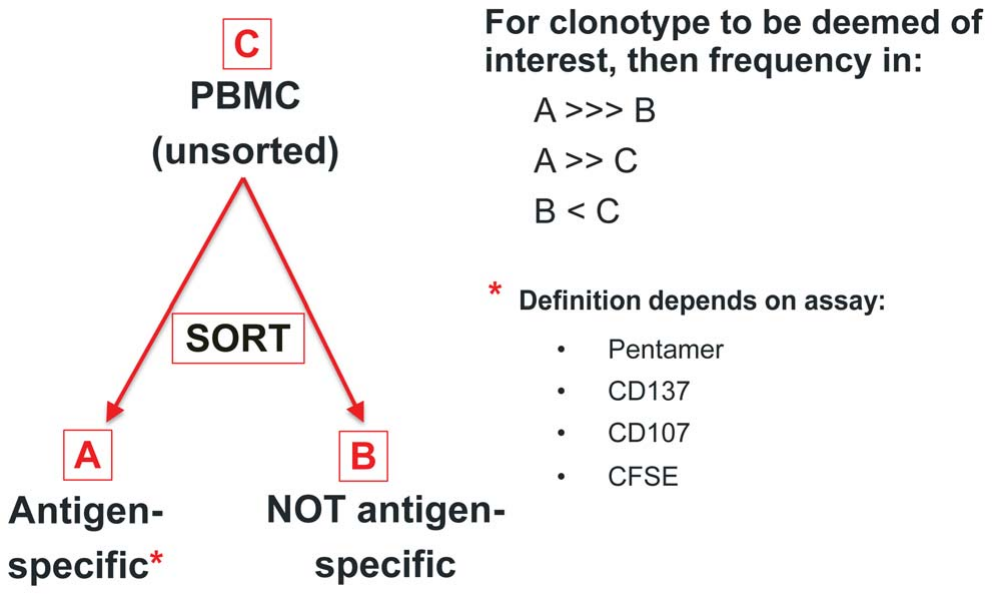
Assay schema.

**Figure 2:**
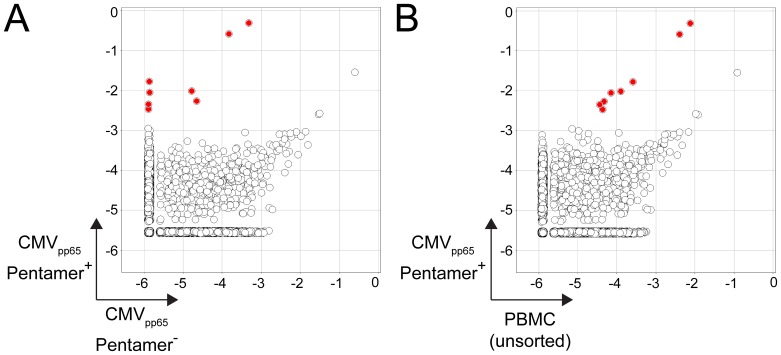
Identification of CMV pp65_495_-specific T cell clonotypes from sorted pentamer^+^ T cells. (**A**) Clonotype frequencies from CMV pp65_495_ pentamer^+^ versus pentamer^−^ CD8^+^ T cells from a characterized CMV responder (outlined in Methods section). The 8 red dots indicate clonotypes greater than 10-fold enriched and exceeding a 20-cell equivalent minimum frequency threshold in the sorted (pentamer^+^) population. (**B**) All 8 clonotypes identified in A are enriched in (unsorted) PBMCs from the same individual. Red dots indicate clonotypes identified in A.

**Figure 3:**
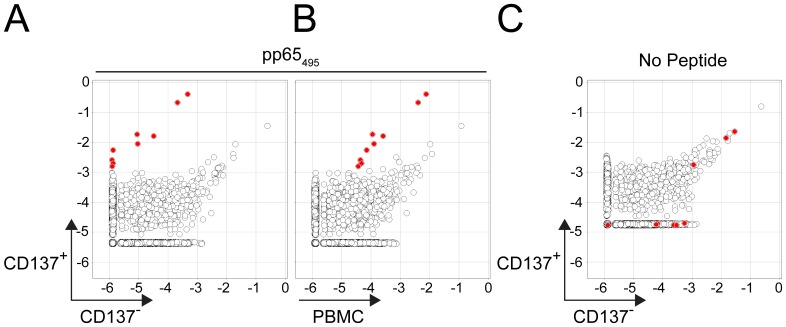
Identification of CMV pp65_495_-specific T cell clonotypes from sorted responding cells following peptide incubation. Clonotype frequencies from sorted responding CD137^+^ cells following CMV pp65_495_ peptide incubation versus either sorted non-responding CD137^−^ cells (**A**) or unsorted PBMCs (**B**). The 9 red dots in A indicate clonotypes greater than 10-fold enriched and exceeding a 20-cell equivalent minimum frequency threshold in the sorted (CD137^+^) population. Red dots in B indicate those clonotypes identified in A. Clonotypes identified in A are not enriched in sorted CD137^+^ cells versus CD137^−^ T cells (**C**) following incubation without peptide.

**Figure 4:**
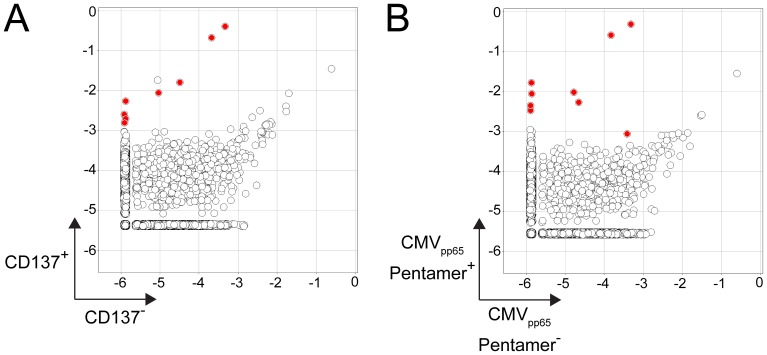
Overlap between clonotypes identified in pentamer and CD137 assays. (**A**) Plot shows clonotype frequencies of the 8 clonotypes (red) identified in the pentamer analyses (outlined in Figure 2A) in the clonotype profiles of CD137^+^ responding cells following CMV pp65_495_ peptide incubation versus sorted non-responding CD137^−^ cells. (**B**) Plot shows clonotype frequencies of the 9 clonotypes (red) identified in the CD137 assay analyses (outlined in Figure 3A) in the clonotype profiles of sorted CMV pp65_495_ pentamer^+^ cells versus pentamer^−^ cells. 8/9 of these clonotypes are overlapping with those identified in A.

**Figure 5:**
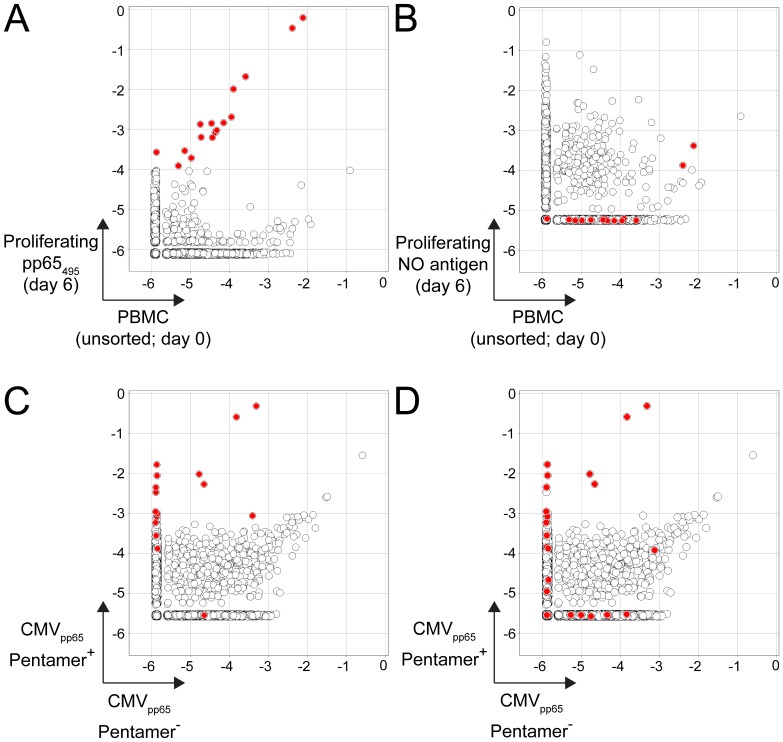
Identification of low-frequency CMV pp65_495_-specific T cell clonotypes following peptide incubation and proliferation. (**A**) Clonotype frequencies from sorted proliferating CD8^+^ T cells following CMV pp65_495_ peptide incubation at day 6 versus fresh unsorted PBMCs. The 16 red dots indicate clonotypes greater than 10-fold enriched and exceeding 1/10,000 minimum frequency threshold in the sorted proliferating cells. (**B**) Clonotype frequencies from sorted proliferating CD8^+^ T cells following incubation without peptide at day 6 versus fresh unsorted PBMCs. Clonotypes in red are those identified in A. (**C**) Clonotype frequencies from CMV pp65_495_ pentamer^+^ versus pentamer^−^ CD8^+^ T cells. Red dots indicate the 16 clonotypes identified in the proliferation assay in A with those clonotypes identified in the CMV pp65_495_ pentamer^+^ versus pentamer^−^ CD8^+^ T cell comparison. (**D**) Clonotype frequencies from CMV pp65_495_ pentamer^+^ versus pentamer^−^ CD8^+^ T cells. Red dots indicate the 25 clonotypes identified in a variant of the proliferation assay described above with those clonotypes identified in the CMV pp65_495_ pentamer^+^ versus pentamer^−^ CD8^+^ T cell comparison. In this assay a pool of 138 overlapping peptides from pp65 was used instead of the single pp65_495_ peptide.

Following clonotype determination, we analyzed the relative frequencies of the clonotypes in the unsorted, antigen-specific and not antigen-specific populations (Figure 1). In many of the figures, we show clonotype frequency comparison between two samples. Clonotypes that are present in sample A but not in sample B are represented to have the frequency of a clonotype with a single read in sample B. Therefore the frequency of the missing clonotype in a sample depends on the sequencing depth of a particular sample (see Figure 2, Figure 3, Figure 4, Figure 5). In these cases where a clonotype is missing in a sample, because we are assigning the frequency of a single read to these clonotypes, we are overestimating the observed frequency. Thus, in the vast majority of these cases, we are likely to be overestimating the real clonotype frequency. A clonotype absent in both samples in Figure 2A for example, is at 10^−5.6^ in the pentamer-positive sample and 10^−5.8^ in pentamer negative sample. Clonotypes absent in both samples appear where the axes intersect. Clonotypes present in one sample but not the other however lie along either the x- or y-axis.

Clonotypes from the antigen-specific T cell analyses were selected based on the following three criteria. First, clonotypes had frequencies that were at least 10-fold increased in sorted antigen-specific versus non-antigen-specific or unsorted cells (see Figure 2A and B example). Second, these clonotypes were also at lower frequency in sorted “not antigen-specific” cells compared to unsorted cells if greater than 1/100,000 in order to avoid sub-sampling error (Poisson noise) associated with very low frequency clonotypes in sorted samples. Third, because of the limited number of input antigen-specific cells after sorting (generally less than <30,000 cells), we applied a greater than ‘20-cell’ equivalent threshold based on the relatively low input number of cells in these samples. This minimum threshold enabled exclusion of clonotypes that appeared enriched in sorted antigen-specific samples but were due only to the presence of one or a few cells in the sample. For example, consider a sorted population of 10,000 pentamer^+^ cells out of a sample with a million T cells. If a single cell with a frequency of 1 per million in the unsorted sample is incidentally sorted in the pentamer^+^ sample, its frequency in the sorted sample will be 1/10,000 and would appear to be 100 fold enriched in the pentamer^+^ sample compared to the unsorted sample. To ameliorate this problem, we required the clonotype to represent 20 cells in the sorted pentamer^+^ sample. Specifically, the log_10_ frequency threshold required in the pentamer^+^ sample was calculated as log_10_(1/(n/20)), where n is the number of input sorted cells for that sample as determined by flow cytometry (See Figure 3A for example −16,281 is number of input sorted cells and the calculated threshold frequency is 10^−2.9^). Those sequences meeting the three criteria outlined above were classified as antigen-specific T cell clonotypes.

## Results

### Identifying CMV pp65-specific CD8 Clonotypes by Combining Sorting and Sequencing of Pentamer^+^ Cells

We assessed whether the combination of sorting and sequencing could identify antigen-specific clonotypes in an individual with a known positive response to a cytomegalovirus (CMV) antigen. First, we paired TCRβ sequencing with a multimer-based immune assay to validate this method for identification of antigen-specific CD8 TCRβ clonotypes. The NLVPMVATV peptide derived from CMV pp65_(495–404)_ is an HLA-A*0201 restricted immunodominant epitope (herein referred to as pp65_495_) that induces responses in CMV-positive individuals. To directly identify T cells specific to this antigen, a commercially available pentamer reagent containing pp65_495_ peptide bound to an MHC molecule was used. In principle, all of the T cells carrying the sequences that bind the pentamer should be detected irrespective of their functional potential. We therefore sought to identify pp65_495_ CD8 T cells by sequencing the TCRβ repertoire of cells that were sorted based on pentamer binding (pentamer^+^). We previously determined that the TCRβ RNA content, as measured by the number of TCRβ RNA molecules per cell, was similar across naïve, activated and memory CD8 T cell subsets (Figure S1).

We obtained frozen PBMCs from an individual with a characterized response to pp65_495_ by ELISPOT assay. Two populations were sorted from this individual: CD8 pentamer^+^ and pentamer^−^ T cells. We sequenced these two populations along with the unsorted PBMC sample and analyzed the relative frequencies of the clonotypes in each population (Figure 1). Three criteria were used to identify pp65_495_-specific TCRβ clonotypes: 1) Clonotypes that are enriched (i.e. have substantially higher frequency) in the pentamer^+^ population compared to the pentamer^−^ population; 2) Clonotypes that are enriched in the pentamer^+^ population compared to the unsorted sample; and 3) Clonotypes that are de-enriched (i.e. have lower frequency) in the pentamer^−^ population compared to the unsorted sample.

We identified 8 clonotypes that are substantially enriched (∼1,000 fold) in the pentamer^+^ compared to the pentamer^−^ population (Figure 2A). We compared the frequency of these clonotypes in the pentamer^+^ and the unsorted populations (Figure 2B). The highest of these clonotypes has a frequency of 0.81% in the unsorted sample, which is consistent with the expected elevated response to pp65_495_ in this individual. However, several of the other clonotypes were present at a level below 10^−4^. The 8 clonotypes are enriched in the pentamer^+^ population by a factor of ∼100 fold compared to their frequency in unsorted PBMC.

### Identifying CMV pp65 Specific CD8 Clonotypes by Combining Sorting and Sequencing of Cells based on Activation Marker Upregulated following Antigen Incubation

We used PBMCs from the same individual to assess whether immune assays that rely on indirect or functional changes in the T cells following antigen stimulation are effective for identification of pp65_495_-specific CD8 TCRβ clonotypes. We tested an assay that is more scalable than a multimer assay, which requires generation of specific reagents for each peptide-HLA type. The assay involves stimulation of PBMCs with pp65_495_ followed by flow cytometry 18 hours after the stimulation to capture cells based on expression of the activation marker CD137. The TCRβ repertoire was amplified and sequenced from sorted CD137^+^ and CD137^−^ cells (Figure 1). The criteria for identification of pp65_495_-specific TCRβ clonotypes with this assay was similar to that used in the pentamer assay. Specifically, pp65_495_-specific TCRβ clonotypes were expected to be present at much higher frequencies in the CD137^+^ population compared to the CD137^−^ population.

We identified 9 clonotypes that are substantially enriched (∼1,000 fold) in the CD137^+^ population compared to the CD137^−^ population (Figure 3A). The frequency of these clonotypes in the unsorted sample ranged from as high as 0.81% to as low as 0.004% (Figure 3B). These clonotypes were enriched in the CD137^+^ population compared to the unsorted PBMC sample by ∼100 fold.

To ensure that these cells were activated due to stimulation with the peptide, we performed a control experiment with no peptide. None of the 9 clonotypes that were enriched with the peptide in the CD137^+^ population enriched following incubation without peptide in CD137^+^ cells (Figure 3C).

All nine antigen-specific clonotypes were present in the sorted CD137^+^ cells following peptide incubation in a second replicate (Figure S2A). Eight antigen-specific clonotypes were identified in replicate 2 (Figure S2A). Seven of these clonotypes overlapped with replicate 1. One additional clonotype was identified in the second replicate that was not identified in the first replicate. Thus, we observed a remarkable overlap of antigen-specific clones identified between the replicates using the CD137 assay.

A similar set of clonotypes was identified using another indirect antigen stimulation assay (CD107) (Figure S3A–S3C), which supports the repeatability of the method using different activation markers. Using the CD107 assay, we tested the reproducibility of the indirect antigen stimulation approach in PBMC from another individual. Specifically, we incubated PBMCs from an HLA A2+ donor with two commercially available and partially overlapping peptide pools (pp65 ‘PepMix’ and CEF+ pools). Independently reported ELISPOT results from this individual indicated a strong positive response to the pp65_495_ single peptide and the CEF+ peptide pool. In this individual, we identified 6 antigen-specific clonotypes following incubation of cells with the pp65 PepMix pool of 138 partially overlapping peptides derived from CMV pp65 (Figure S4A). We identified 4 antigen-specific clonotypes following incubation of cells with the CEF+ peptide pool (Figure S4B). Three of the clonotypes identified with the pp65 pool were also identified with the CEF+ pool. These results indicate that 75% (3 of 4) of the antigen-specific clonotypes identified with the CEF+ peptide pool in the CD107 assay are likely specific for pp65_495_. This data supports the reproducibility of this method in multiple individuals for identification of antigen-specific clonotypes.

### Overlap between Clonotypes Identified in Pentamer and CD137 Assays

We compared the specific clonotypes identified by the pentamer and CD137 assays and found a substantial amount of overlap. All 8 clonotypes that were identified with the pentamer assay were also identified by CD137 assay (Figure 4A and Table 1). An additional clonotype was identified by the CD137 assay but not the pentamer assay (Figure 4A). As evident in Figure 4B, this clonotype is slightly enriched in the pentamer^+^ population but not to the same extent as the other clonotypes and thus did not meet the selection criteria. The overlap between clonotypes identified following direct identification of cells with an MHC class I pentamer and indirect identification of antigen-specific cells following a functional response to antigen validates the accuracy of the sequencing-based method for identification of antigen-specific clonotypes.

**Table 1:** Summary of CMV pp65_495_-specific CD8^+^ T cell clonotypes identified in each immune assay.

		Clonotype Frequency (Log_10_)					
CDR3 ID	Clone_Protein_Sequence	Pentamer^−^	Pentamer^+^	CD137^−^	CD137^+^	PBMC(unsorted)	Proliferating(day 6)
1	NQTSLYFCASRLLAGGDNEQFFGPGTRLTV	**−**5.87	**−**2.934	**−**5.893	**−**3.015	**−5.124**	**−3.519**
2	SQTSVYFCASSAYTGTVYGYTFGSGTRLTV	**−**5.87	**−**2.982	−5.893	−3.639	**−4.999**	**−3.7**
3	PSQTSVYFCASSLSTGTSYGYTFGSGTRLTV	−5.87	−3.06	−5.893	−3.199	**−4.726**	**−3.18**
4	DSAMYFCASSEALLGRANYGYTFGSGTRLTV	**−5.87**	**−2.02**	**−5.893**	**−2.233**	**−4.162**	**−2.807**
5	RDSAMYRCASSLAPGTTNEKLFFGSGTQLSV	**−5.87**	**−2.487**	**−5.893**	**−2.615**	**−4.346**	**−3.099**
6	GDSALYFCASSPGQTFSNQPQHFGDGTRLSI	−4.639	−5.551	−5.893	−3.299	**−5.3**	**−3.918**
7	SQTSVYFCASSPLTGTGVYGYTFGSGTRLTV	**−3.29**	**−0.3054**	**−3.303**	**−0.3877**	**−2.091**	**−0.206**
8	SQTSVYFCASSPTTGTGNYGYTFGSGTRLTV	−5.87	−3.223	−5.893	−3.023	**−4.726**	**−2.868**
9	EDSAVYLCASSSSFPGGGNEQFFGPGTRLTV	**−4.666**	**−2.243**	**−5.893**	**−2.671**	**−4.346**	**−2.996**
10	PSQTSVYFCASSYATGASYGYTFGSGTRLTV	**−5.87**	**−1.764**	**−4.495**	**−1.777**	**−3.593**	**−1.666**
11	PSQTSVYFCASSYQTGAAYGYTFGSGTRLTV	**−4.791**	**−2.045**	**−5.048**	**−2.081**	**−3.911**	**−2.027**
12	PSQTSVYFCASSYQTGASYGYTFGSGTRLTV	**−3.824**	**−0.6073**	**−3.676**	**−0.6908**	**−2.395**	**−0.4888**
13	GDSALYFCASTLLGGAGYNEQFFGPGTRLTV	−3.4	−3.03	**−5.048**	**−1.709**	**−3.943**	**−2.669**
14	GGLRRVSLCQQLLFSGGGNEQFFGPGTRLTV	**−5.87**	**−2.339**	**−5.893**	**−2.797**	**−4.425**	**−3.201**
15	RLIHYSVGAGITDQGEVPNGYTFGSGTRLTV	−5.87	−3.573	−4.814	−3.291	**−4.455**	**−2.871**
16	RLIHYSVGAGITDQGEVPYGYTFGSGTRLTV	−5.87	−3.898	−5.893	−3.494	**−5.902**	**−3.595**

The table lists all 16 clonotype sequences and corresponding frequencies in each of the sorted populations for each of the four immune assays as well as clonotype frequencies in fresh unsorted PBMCs. Frequencies in bold font indicate the clonotypes that met the selection criteria for that assay. Frequencies in normal font did not meet the selection criteria for that assay.

### Identifying CMV pp65 Specific Clonotypes by Combining Sorting and Sequencing of Cells based on Proliferation following Antigen Incubation

We assessed the utility of a third functional assay for identification of antigen-specific clonotypes by combining capture of proliferating cells following incubation with pp65_495_ peptide and repertoire sequencing. We reasoned that employing a proliferation assay could increase the sensitivity of detection of antigen-specific T cells, as even a single cell can potentially increase in frequency substantially to allow for identification. As shown in Figure 1, we labeled cells with CFSE and incubated them with either pp65_495_ or no peptide for 6 days. Proliferating CD8 cells were then sorted based on dilution of CFSE. pp65_495_-specific clonotypes were identified based on their relative frequency in the CFSE^low^ population compared to that of fresh unsorted PBMCs.

We identified 16 clonotypes that were substantially increased in the CFSE^low^ population, and the frequency of some of the identified clonotypes was below 10^−5^ (Figure 5A). We used an identical proliferation assay that lacked the peptide as a control. None of the 16 clonotypes identified by the proliferation assay were enriched in the CFSE^low^ population when no peptide was used (Figure 5B).

We then confirmed the authenticity of the clonotypes that were enriched in the proliferation assay. If these were truly pp65_495_-specific clonotypes, it was anticipated we would find them enriched in the pentamer^+^ (or CD137^+^) population, but the low number of cells did not allow us to identify them confidently by the pentamer (or CD137^+^) analysis. Indeed this phenomena was observed (Figure 5C and Table 1). Essentially all of the clonotypes identified by the proliferation assay were enriched by the other assays, which was consistent with their identification as authentic pp65_495_-specific clonotypes and not resulting from bystander activation.

One advantage to using indirect immune monitoring assays compared to pentamer reagents is the ability to assess responses to more than one peptide antigen at the same time. We therefore used a pool of 138 peptides spanning the entire pp65 protein (herein referred to as pp65_pool_) to stimulate PBMCs in the proliferation assay to identify pp65_pool_-specific T cells. Repertoire analysis of proliferating cells following pp65_pool_ incubation enabled identification of 25 clonotypes. Of these 25 clonotypes identified using the pp65_pool_, 12 of these were also deemed antigen-specific with the single pp65_495_ peptide (Figure S2B), demonstrating the repeatability of the approach.

Seven of eight clonotypes identified by the pentamer assay were identified in the pp65_pool_ proliferation assay, demonstrating that the use of peptide pool does not substantially decrease sensitivity. In addition the proliferation assay with the pp65_pool_ enabled identification of additional clonotypes that are presumably specific to other peptides within the pool. Most of the additional clonotypes identified with the pp65_pool_ were not enriched in the pentamer^+^ population (Figure 5D) consistent with them being not specific to the pp65_495_ peptide.

### Sequence Comparisons of Identified pp65 Specific TCRβ Clonotypes

After identifying the pp65_495_-specific clonotypes, we analyzed the clonotype sequences and found obvious similarities between some of the clonotype CDR3 amino acid sequences. Specifically, three sets of CDR3 amino acid sequences showed remarkable similarity (with 3 or fewer amino acid differences) out of the 16 clonotypes identified with these assays in this individual (summarized in Table 1). For example, among the 8 clonotypes identified as pentamer^+^, three encoded CDR3s that were very similar to each other (3 amino acid difference or less; CDR3 IDs 10, 11 and 12 in Table 1). In addition, among the clonotypes that were identified only by the proliferation assay there was another clonotype that was similar (2 amino acid difference; CDR3 IDs 7 and 8 in Table 1) to one of the 8 clonotypes identified by the pentamer^+^ assay (Table 1). An additional pair of clonotypes among the set identified with the proliferation assay differed by three amino acids in the CDR3 region (CDR3 IDs 2 and 3 in Table 1). Many of the CDR3 amino acid differences described above occur between either conserved residues or similarly sized polar uncharged and hydrophobic side chain residues. These results are consistent with previous reports in which antigen-specific T cells were sorted and CDR3β sequences determined [25]–[30]. This sequence-level data adds to the validation of these low frequency clonotypes identified by the proliferation assay.

We also compared clonotype CDR3 amino acid sequences to those previously published. One of the clonotypes identified in the assays described above was identical to a previously published pp65_495_-specific CDR3 sequence (CDR3 sequence CASSYQTGAAYGYTF; CDR3 ID 11 in Table 1) [31], [32]. Moreover, the top clonotype identified with the CD107 assay in the other individual tested in this study was identical to a different published pp65_495_-specific sequence (CDR3 sequence CASSLEGYTEAFFG; see top clone in red in Figure S4A and B) [33], [34], thus illustrating the ease with which this approach enables identification of public CDR3 sequences. Together, these data provide validation at the sequence-level of the antigen-specific CD8 clonotypes identified using peptide antigens.

## Discussion

The past decade has seen many advances in immune monitoring techniques, and a wide portfolio of immune assays can be used to identify and monitor antigen-specific T cell responses. Despite these advances, current immune assays are limited by their sensitivity and inability to discriminate the component T cells, or clonotypes, that contribute to an immune response. We developed a high throughput sequencing-based method which can identify individual clonotypes based on their unique T cell receptor rearrangement [16], [24]. This sequencing method can be used to characterize the diversity of clonotypes that contribute to an immune response; however, it is limited in its ability to identify clonotypes with specific reactivity to a particular antigen. Here we combined the sequencing method with existing immune assays to characterize immune responses following exposure to CMV-specific antigens. In this novel approach, immune assays provide qualitative antigen-specific information, while sequencing reveals the individual clonotypes that contribute to an immune response with great sensitivity and quantitative accuracy.

We validated this approach using three types of immune assays: pentamers, which enable identification of specific TCRs, and two types of functional assays (CD137 and proliferation) which enable identification of cells responding to antigen following incubation to identify CMV-specific clonotypes. The antigen-specific clonotypes identified by each immune assay were largely overlapping and consistent among methodologies. Further validation of this method was provided by sequence-level analysis of the resulting clonotypes. These results demonstrate the robustness of our approach and highlight the fact that sequencing can be effectively combined with multiple immune assays for identification of antigen-specific T cells.

We observed a substantial level of clonotypic diversity in response to antigen stimulation. Using the proliferation assay, we observed 16 clonotypes that were responsive to pp65 antigen stimulation. Many of these pp65-specific clonotypes were present at frequencies below 10^−5^ and, therefore, were undetectable using standard flow-cytometric methods. This data suggests that the immune response is comprised of a diverse set of clones many at very low frequency that synergistically contribute to an immune response. Thus, the combination of immune assays and sequencing depicts the richness and diversity of the immune response, at a level that is not possible using standard immune assays alone. Once clonotypes are identified as antigen-specific using the combination of immune assays and sequencing, their frequency can then be tracked over time using sequencing alone.

In summary, the combination of repertoire sequencing and immune assays represents a powerful, high-resolution approach for characterization of an immune response. While this study provides a proof of principle for the identification of antigen-specific clonotypes in the CMV model system, the methods articulated in this work are applicable to many fields of medicine. An enhanced knowledge of how T cell-mediated immunity is established and maintained at the clonal level may inform strategies for improved vaccine and immunotherapy protocols. Moreover, immune cell receptor sequencing could ultimately form the basis for diagnostic, prognostic and disease-monitoring tools for immune-mediated disorders.

## Supporting Information

Figure S1 **Graph shows the number of TCRβ RNA molecules per cell from naïve (CD8^+^CD69^−^CCR7^+^CD45RA^+^), activated (CD8^+^CD69^+^) and memory (CD8^+^CD69^−^CCR7^−^CD45RA^−^, CD8^+^CD69^−^CCR7^+^CD45RA^−^ and CD8^+^CD69^−^CCR7^−^CD45RA^+^) CD8 T cell populations sorted from three individuals.**
(TIF)

Figure S2 **Clonotype overlap between replicate experiments. (A)** CD137 assay replicate. Clonotype frequencies from sorted responding CD137^+^ cells following CMV pp65_495_ peptide incubation versus sorted non-responding CD137^−^ cells in a second replicate experiment. The nine clonotypes indicated in red were those deemed antigen-specific in the first replicate and shown in Figure 3A. The two lowest frequency clonotypes in red in replicate 2 did not meet the criteria for antigen-specificity. The single clonotype indicated in blue was identified in replicate 2 but not replicate 1. Seven clonotypes identified in replicate 1 were also identified in replicate 2. **(B)** Proliferation assay partial replicate: single versus peptide pool comparison. Clonotype frequencies from sorted proliferating CD8^+^ T cells following incubation with the CMV pp65_pool_ at day 6 versus fresh unsorted PBMCs. The 16 red dots indicate those clonotypes identified with the single CMV pp65_495_ peptide and shown in Figure 5A. 12 out of 16 clonotypes identified with the single peptide met the antigen-specific clonotype selection criteria (>10-fold enriched AND >1/10,000 minimum frequency threshold in the proliferating cell sample) in the peptide pool experiment. Thirteen additional clonotypes indicated in blue were identified with the peptide pool but not with the single CMV pp65_495_ peptide.
(TIF)

Figure S3 **Identification of CMV pp65_495_-specific T cell clonotypes from sorted responding cells following peptide incubation.** Clonotype frequencies from sorted responding CD107^+^ cells following CMV pp65_495_ peptide incubation versus either sorted non-responding CD107^−^ cells **(A)** or unsorted PBMCs **(B)**. The 7 red dots in A indicate clonotypes greater than 10-fold enriched and exceeding a 20-cell equivalent minimum frequency threshold in the sorted (CD107^+^) population. Red dots in B indicate those clonotypes identified in A. Clonotypes identified in A are not enriched in sorted CD107^+^ cells versus CD107^−^ T cells **(C)** following incubation without peptide.
(TIF)

Figure S4 **Antigen-specific clonotypes identified following incubation with partially overlapping peptide pools. (A)** Clonotype frequencies from sorted responding CD107^+^ cells following pp65 ‘PepMix’ peptide pool incubation versus sorted non-responding CD107^−^ cells. The 6 red dots indicate clonotypes greater than 10-fold enriched and exceeding a 20-cell equivalent minimum frequency threshold in the sorted (CD107^+^) population. **(B)** Clonotype frequencies from sorted responding CD107^+^ cells following CEF+ peptide pool incubation versus sorted non-responding CD107^−^ cells. The 4 red dots indicate clonotypes greater than 10-fold enriched and exceeding a 20-cell equivalent minimum frequency threshold in the sorted (CD107^+^) population. Three of the clonotypes identified in A above are identical to those identified in B. One of the CMV pp65-derived peptides in the CEF+ peptide pool likely overlaps with peptide(s) derived from the pp65 ‘PepMix’. This PBMC donor exhibited a very strong response to CMV pp65_495_ by ELISPOT analyses.
(TIF)
