# Supplementary figures and images for: Combining Next-Generation Sequencing and Immune Assays: A Novel Method for Identification of Antigen-Specific T Cells
Source: PLoS One. 2013 Sep 19;8(9):e74231. doi: 10.1371/journal.pone.0074231 (PMC3778005; doi:10.1371/journal.pone.0074231)

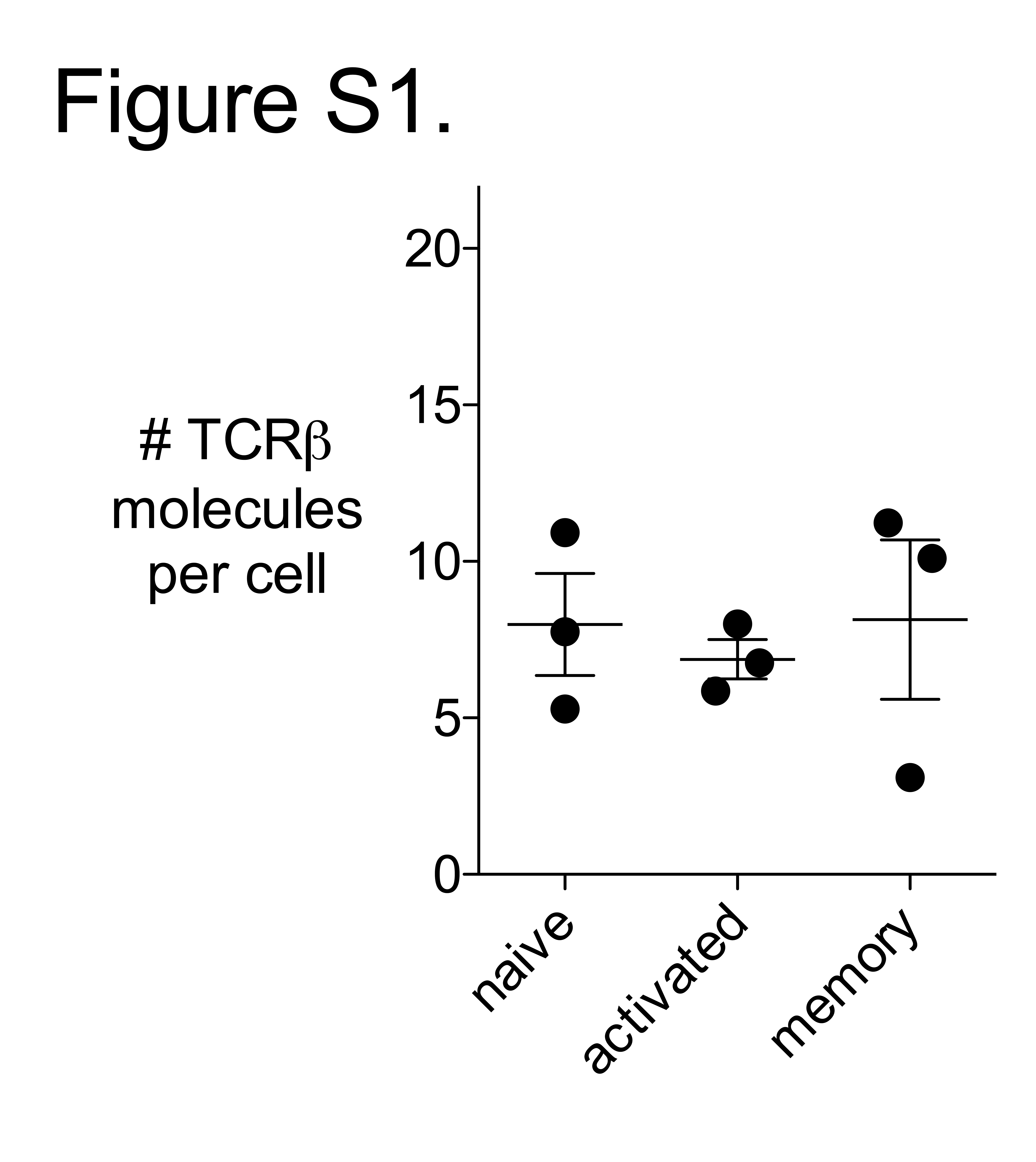

Supplement: Figure S1 — Graph shows the number of TCRβ RNA molecules per cell from naïve (CD8+CD69−CCR7+CD45RA+), activated (CD8+CD69+) and memory (CD8+CD69−CCR7−CD45RA−, CD8+CD69−CCR7+CD45RA− and CD8+CD69−CCR7−CD45RA+) CD8 T cell populations sorted from three individuals. (TIF) [file pone.0074231.s001.tif]

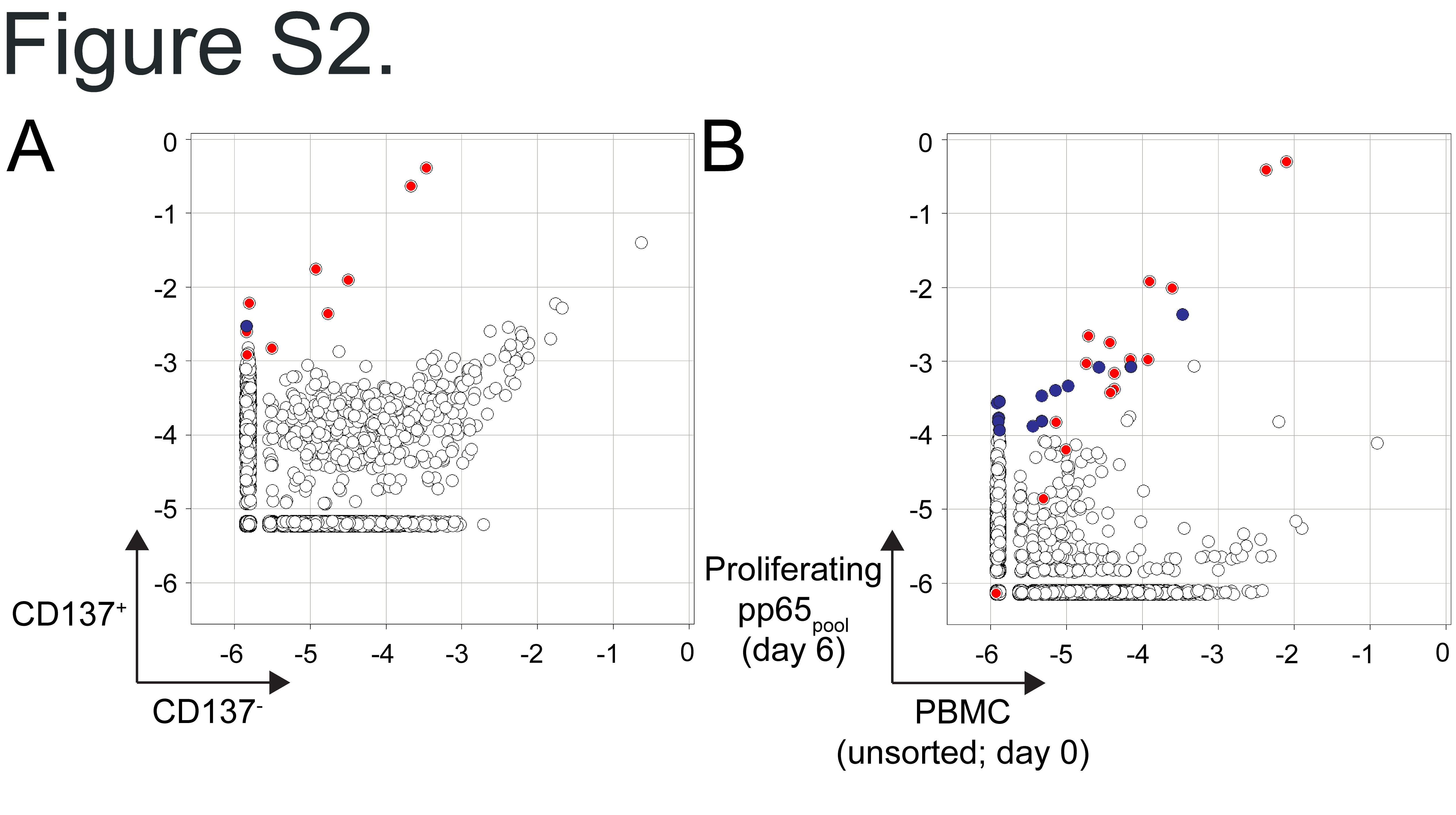

Supplement: Figure S2 — Clonotype overlap between replicate experiments. (A) CD137 assay replicate. Clonotype frequencies from sorted responding CD137+ cells following CMV pp65495 peptide incubation versus sorted non-responding CD137− cells in a second replicate experiment. The nine clonotypes indicated in red were those deemed antigen-specific in the first replicate and shown in Figure 3A. The two lowest frequency clonotypes in red in replicate 2 did not meet the criteria for antigen-specificity. The single clonotype indicated in blue was identified in replicate 2 but not replicate 1. Seven clonotypes identified in replicate 1 were also identified in replicate 2. (B) Proliferation assay partial replicate: single versus peptide pool comparison. Clonotype frequencies from sorted proliferating CD8+ T cells following incubation with the CMV pp65pool at day 6 versus fresh unsorted PBMCs. The 16 red dots indicate those clonotypes identified with the single CMV pp65495 peptide and shown in Figure 5A. 12 out of 16 clonotypes identified with the single peptide met the antigen-specific clonotype selection criteria (>10-fold enriched AND >1/10,000 minimum frequency threshold in the proliferating cell sample) in the peptide pool experiment. Thirteen additional clonotypes indicated in blue were identified with the peptide pool but not with the single CMV pp65495 peptide. (TIF) [file pone.0074231.s002.tif]

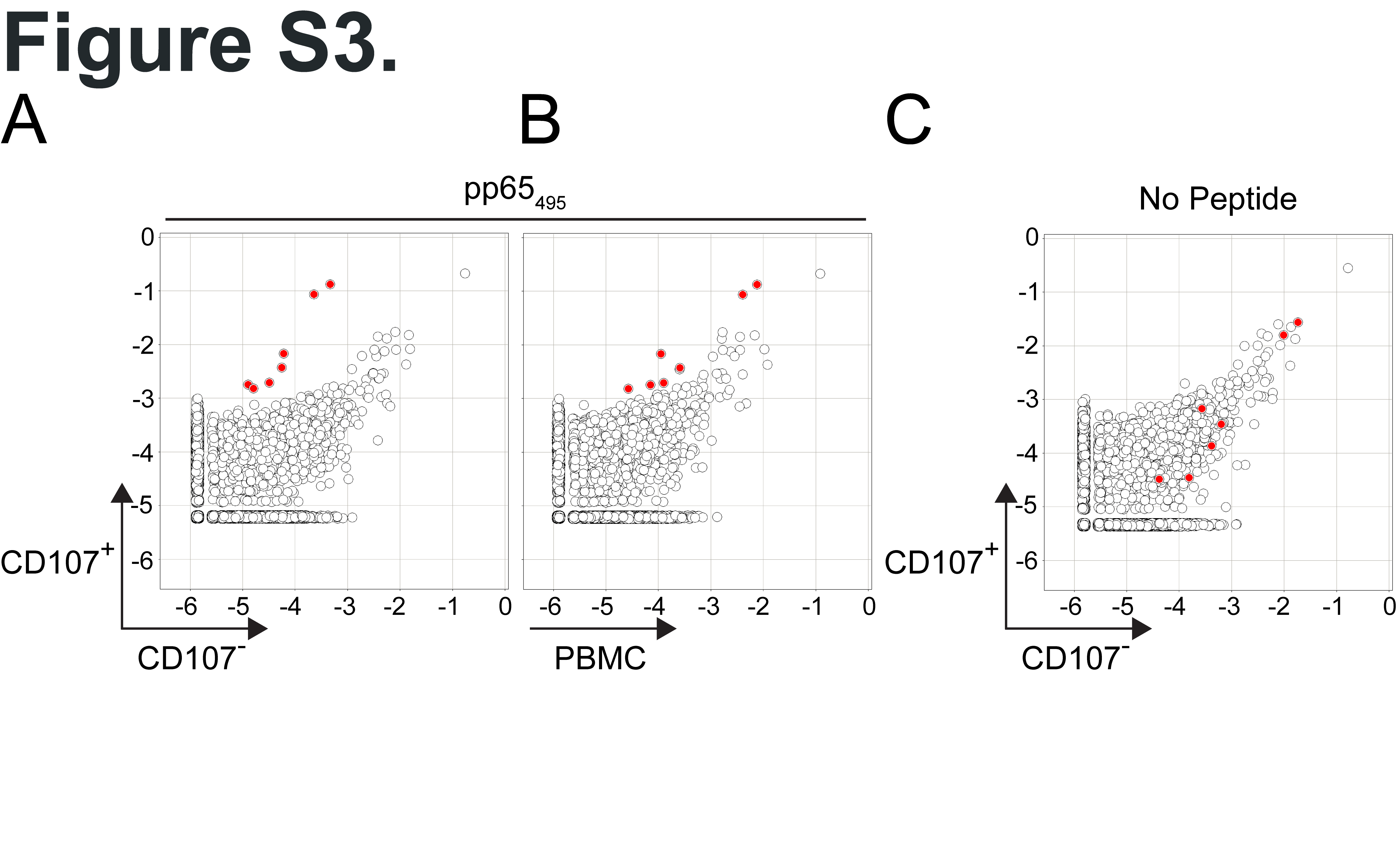

Supplement: Figure S3 — Identification of CMV pp65495-specific T cell clonotypes from sorted responding cells following peptide incubation. Clonotype frequencies from sorted responding CD107+ cells following CMV pp65495 peptide incubation versus either sorted non-responding CD107− cells (A) or unsorted PBMCs (B). The 7 red dots in A indicate clonotypes greater than 10-fold enriched and exceeding a 20-cell equivalent minimum frequency threshold in the sorted (CD107+) population. Red dots in B indicate those clonotypes identified in A. Clonotypes identified in A are not enriched in sorted CD107+ cells versus CD107− T cells (C) following incubation without peptide. (TIF) [file pone.0074231.s003.tif]

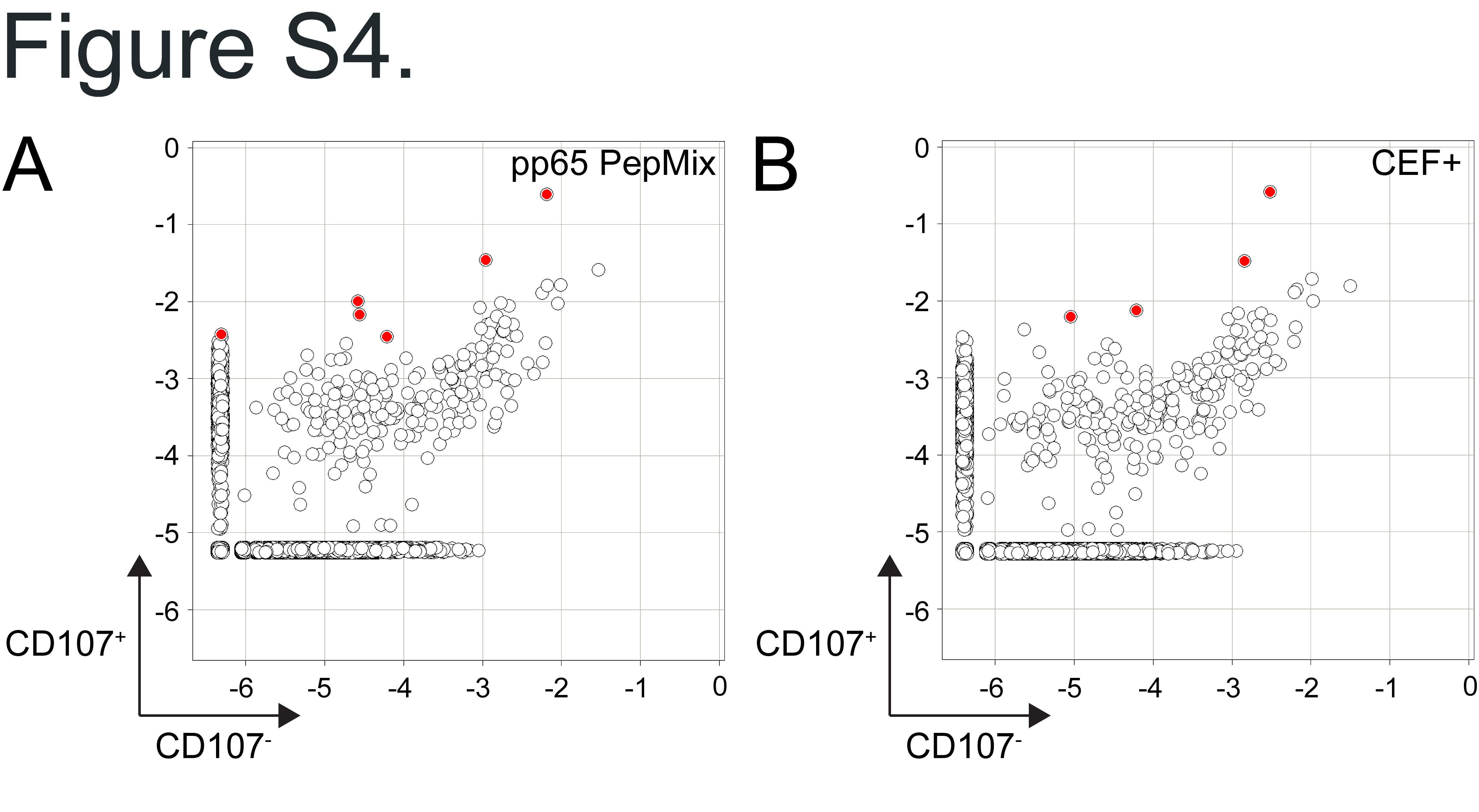

Supplement: Figure S4 — Antigen-specific clonotypes identified following incubation with partially overlapping peptide pools. (A) Clonotype frequencies from sorted responding CD107+ cells following pp65 ‘PepMix’ peptide pool incubation versus sorted non-responding CD107− cells. The 6 red dots indicate clonotypes greater than 10-fold enriched and exceeding a 20-cell equivalent minimum frequency threshold in the sorted (CD107+) population. (B) Clonotype frequencies from sorted responding CD107+ cells following CEF+ peptide pool incubation versus sorted non-responding CD107− cells. The 4 red dots indicate clonotypes greater than 10-fold enriched and exceeding a 20-cell equivalent minimum frequency threshold in the sorted (CD107+) population. Three of the clonotypes identified in A above are identical to those identified in B. One of the CMV pp65-derived peptides in the CEF+ peptide pool likely overlaps with peptide(s) derived from the pp65 ‘PepMix’. This PBMC donor exhibited a very strong response to CMV pp65495 by ELISPOT analyses. (TIF) [file pone.0074231.s004.tif]
